# Allergen-Specific Antibodies Regulate Secondary Allergen-Specific Immune Responses

**DOI:** 10.3389/fimmu.2018.03131

**Published:** 2019-01-17

**Authors:** Julia Eckl-Dorna, Sergio Villazala-Merino, Birgit Linhart, Alexander V. Karaulov, Yury Zhernov, Musa Khaitov, Verena Niederberger-Leppin, Rudolf Valenta

**Affiliations:** ^1^Department of Otorhinolaryngology, Medical University of Vienna, Vienna, Austria; ^2^Division of Immunopathology, Department of Pathophysiology and Allergy Research, Center for Pathophysiology, Infectiology and Immunology, Medical University of Vienna, Vienna, Austria; ^3^Laboratory of Immunopathology, Department of Clinical Immunology and Allergy, Sechenov First Moscow State Medical University, Moscow, Russia; ^4^NRC Institute of Immunology FMBA of Russia, Moscow, Russia

**Keywords:** allergy, allergen, antibody, facilitated allergen presentation (FAP), T cell response, allergen-specific immunotherapy, super-crosslinking, IgE^+^ memory B cell

## Abstract

Immunoglobulin E (IgE)-associated allergy is the most common immunologically-mediated hypersensensitivity disease. It is based on the production of IgE antibodies and T cell responses against *per se* innocuous antigens (i.e., allergens) and subsequent allergen-induced inflammation in genetically pre-disposed individuals. While allergen exposure in sensitized subjects mainly boosts IgE production and T cell activation, successful allergen-specific immunotherapy (AIT) induces the production of allergen-specific IgG antibodies and reduces T cell activity. Under both circumstances, the resulting allergen-antibody complexes play a major role in modulating secondary allergen-specific immune responses: Allergen-IgE complexes induce mast cell and basophil activation and perpetuate allergen-specific T cell responses via presentation of allergen by allergen presenting cells to T cells, a process called IgE-facilitated antigen presentation (FAP). In addition, they may induce activation of IgE memory B cells. Allergen-induced production of specific IgGs usually exerts ameliorating effects but under certain circumstances may also contribute to exacerbation. Allergen-specific IgG antibodies induced by AIT which compete with IgE for allergen binding (i.e., blocking IgG) inhibit formation of IgE-allergen complexes and reduce activation of effector cells, B cells and indirectly T cells as FAP is prevented. Experimental data provide evidence that by binding of allergen-specific IgG to epitopes different from those recognized by IgE, allergen-specific IgG may enhance IgE-mediated activation of mast cells, basophils and allergen-specific IgE^+^ B cells. In this review we provide an overview about the role of allergen-specific antibodies in regulating secondary allergen-specific immune responses.

## Background

Approximately 30% of the world population suffer from immunoglobulin E (IgE)-associated allergy, the most common hypersensitivity disease. Besides environmental factors also genetic factors are very important for the development of IgE responses. They include genetic factors promoting Th2 immune responses and IgE production in general as well as genetic restriction of allergen-specific immune responses (1–3). Allergic patients suffer from a broad variety of symptoms such as rhinoconjunctivitis, asthma, skin inflammation, gastrointestinal symptoms, and life-threatening allergic shock. Allergen-specific antibodies play a major role in development, maintenance, and treatment of allergic diseases (3, 4). During early-phase responses to allergen encounter, crosslinking of surface receptor bound IgE on mast cells and basophils by its cognate allergen induces activation of these cells (5). This signaling cascade finally results in mediator release causing symptoms of the immediate inflammatory responses. Despite its prominent role in mediating early effector cell activation, IgE may also contribute to late phase reactions in allergy. Antigen presenting cells (APCs) can bind and internalize allergen-IgE complexes via IgE receptors and thereby enhance allergen-specific T cell activation (6). The latter role of IgE in regulating T cell responses is supported not only by *in vitro* experiments but also by the observation that anti-IgE treatment alleviates late phase reactions in allergic asthmatic patients (7). The effect of treatment-induced reduction of IgE-meditated T cell activation (7) may also act in concert with a decrease in mast cell/basophil activation (8) and associated reduced release of inflammatory cytokines (9, 10) leading to an amelioration in late phase reactions upon anti-IgE treatment.

So far, allergen-specific immunotherapy (AIT) is the only disease-modifying treatment in allergy with long lasting clinical effects and modulation of the allergic immune response (11, 12). The mechanisms by which AIT effectively reduces allergic inflammation includes changes in cellular as well as humoral responses to allergen contact (13–16). One cardinal feature of successful AIT is the induction of allergen-specific IgG production. In AIT treated patients, a rise in allergen-specific IgG, of the IgG_4_ and IgG_1_ subclass, is observed both in serum (17–19) as well as locally for example in nasal secretions (20, 21). AIT-induced allergen specific IgG4 antibodies have received particular attention because they seem to be responsible for the sustained effects of this treatment (22). Though IgG_4_ accounts for only 4% of total IgG in healthy individuals, it can represent up to 75% of total IgG in subjects undergoing allergen immunotherapy (23). Importantly, allergen-IgG_4_ immune complexes are non-inflammatory because IgG_4_ does not activate complement. Moreover, it has been suggested that IgG_4_ can form bispecific and functionally monovalent antibodies by exchange of Fab arms under certain conditions (24, 25). Ideally, IgGs induced during AIT are induced to block the binding of IgE to the allergen either by occupying IgE epitopes or parts thereof and/or by steric hindrance. They compete with IgE for the binding to the allergen and are thus termed “blocking antibodies” (4, 26). By blocking binding of IgE to the allergen, they may on the one hand inhibit boosting of IgE production by B cells as well as mast cell and basophil activation but they can also block the presentation of allergen by IgE-mediated allergen presentation to T cells (13, 27).

## Role of Allergen-Specific Antibodies in the Natural Course of the Disease

**Figure 1:**
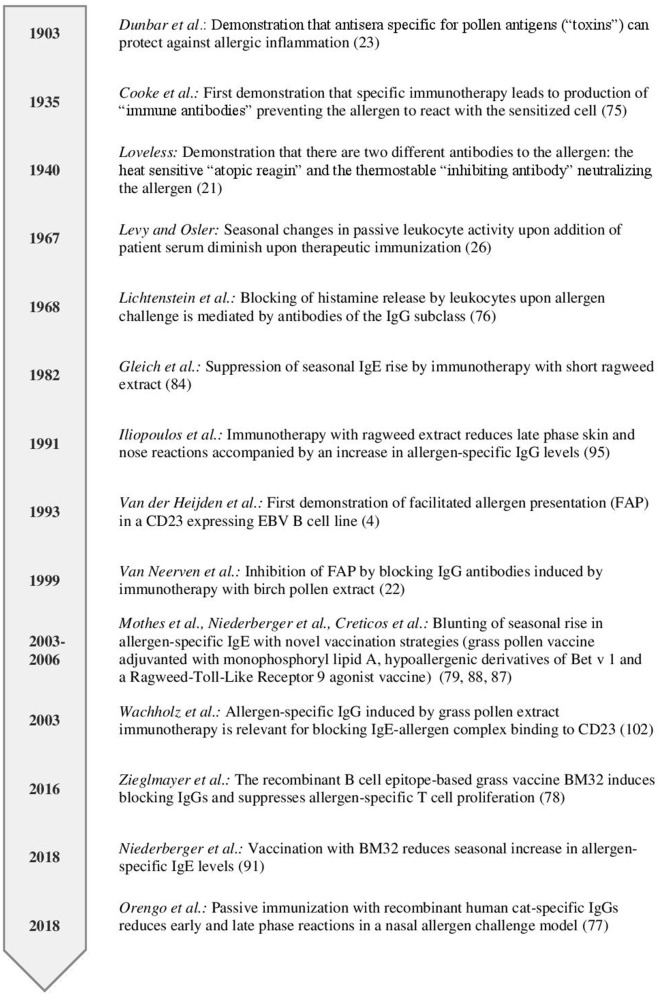
Timeline highlighting studies investigating the role of antibodies in regulating secondary immune responses.

Already in 1903, long before allergy was recognized as an immunologically-mediated hypersensitivity disease, Dunbar demonstrated that allergic reactions in patients could be ameliorated when the disease-causing allergens were neutralized with an allergen-specific antiserum (28) (Figure 1). IgE was identified as a new class of immunoglobulins responsible for allergic reactions in 1966 (29) and became detectable in blood by serology in 1967 (30). In the same year, Levy and Osler reported that the “reagenic reactivity” mediated by IgE in serum of ragweed pollen allergic patients as measured by passive leukocyte sensitivity was lowest before the ragweed season and highest after the season during the autumn months (31) (Figure 1). Later, the reagenic activity was attributed to allergen-specific IgE and rises in allergen-specific serum IgE levels were measured after allergen exposure (32, 33).

Receptor bound IgE can persist on mast cells in tissues for weeks if not months (34). This is supported by the fact that non-allergic recipients of solid organ transplants can exhibit allergic reactions mediated by mast cell-fixed IgE transferred from an allergic donors months after the transplantation (35). Furthermore, anti-IgE treatment has an immediate effect on free IgE levels, but downregulation of high affinity receptors of IgE takes several weeks indicating long survival of IgE in a receptor bound form (36). In contrast free serum IgE has a rather short half-life of 2–3 days (37, 38) and needs to be replenished constantly to sustain allergen-specific IgE levels also in the absence of allergenic stimulation. The mechanisms underlying secondary allergen-specific IgE responses as well as the existence of memory IgE^+^ B cells and long-lived IgE plasma cells in human subjects are still debated (3, 39). Some studies employing flow cytometry suggest existence of both IgE^+^ memory B cell as well as plasma cells in the blood (40–43). However it needs to be borne in mind that in these flow cytometric analyses co-staining for the low affinity receptor for IgE, CD23, that is known to be expressed both on naïve as well as on memory B cells, albeit at a lower level, (44) and to bind IgE, was not performed. Another limitation is that only the minority of IgE is produced in the blood (45) and that the quest for IgE memory should probably be extended to other tissues bearing the environment for memory cell propagation and homing of plasma cells (3, 46). In this context, local IgE production has been observed in samples derived from nasal mucosa of atopic patients (47, 48). Indirect evidence for the existence of IgE memory comes from two major observations: Firstly atopic disposition was transferred by bone marrow transplantation and was still detectable 2 years afterwards (49). Secondly, patients undergoing controlled nasal provocation with defined amounts of recombinant allergens or extract clearly show a rise in allergen-specific IgE levels while allergen-specific IgG and IgM levels remained unchanged indicating the presence of IgE^+^ memory B cells eventually residing in the nasal mucosa that can be activated upon nasal allergen contact (33, 50–52).

Allergen-specific antibodies of the IgG class are present both in non-atopic as well as atopic subjects (53) but only a weak correlation between allergen-specific IgE and IgG responses has been observed (54). This divergence of IgE and IgG responses is already evident in early childhood suggesting that non-sequential class switch to IgE and IgG occurs independently (55, 56). While the IgG repertoire seems to be broader and starting with responses to foodborne molecules in early childhood, IgE is mostly directed to airborne molecules with a sequence and prevalence hierarchy. These findings indicate that IgE and IgG are raised toward different epitopes in atopic subjects. Whether allergen-specific IgG antibodies which recognize different epitopes on allergens than IgE antibodies and hence do not represent blocking IgG antibodies have functional roles in allergic patients is not well-investigated. It is however quite possible that the concurrent presence of allergen-specific polyclonal IgG in allergic patients may influence the secondary allergen-specific immune response at different levels as discussed below.

### Antibody-Mediated Facilitated Allergen Presentation to T Cells

**Figure 2:**
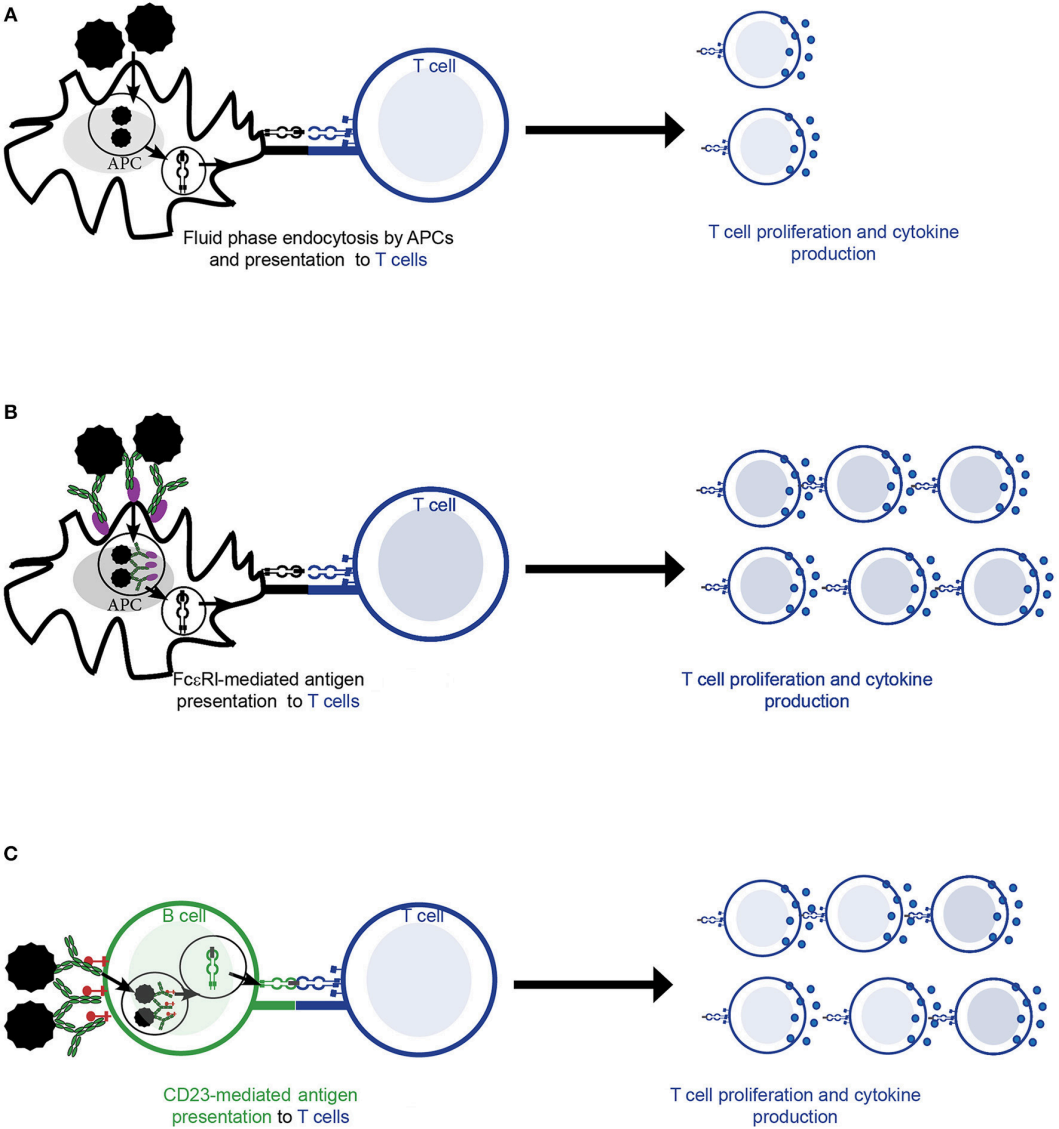
Allergen-specific T cell activation by antigen presenting cells (APCs). **(A–C)** Allergen is taken up by APCs and subsequently presented to allergen-specific T cell cells in the context of MHCII and co-stimulatory molecules. This is followed by T cell activation resulting in allergen-specific proliferation and cytokine production. **(A)** Fluid phase endocytosis of allergen by APCs. **(B)** Allergen-IgE complexes are bound and internalized by the high affinity receptor for IgE (FcεRI) on the surface of APCs (e.g., dendritic cells). **(C)** Allergen-IgE complexes are bound and internalized via the low affinity receptor for IgE (CD23) present mainly on the surface of B cells.

Different pathways and cell types can mediate the activation of allergen-specific T cells in the context of allergy. T cells can be activated by presentation of allergen by antigen-presenting cells (APCs) that has previously been internalized by fluid phase endocytosis (Figure 2A). However, in the context of allergy, receptor-mediated internalization of allergen-IgE complexes via high (FcεRI) affinity (Figure 2B) and low (CD23) affinity (Figure 2C) receptors for IgE by APCs- a process called facilitated antigen presentation (FAP)—has been shown to stimulate allergen-specific T cell proliferation more efficiently, in particular at low concentrations of allergen as they occur *in vivo* in allergic patients (6, 57–60). In this line, presentation via CD23-mediated FAP requires 100–1,000 times lower allergen concentration than presentation via fluid phase endocytosis (6, 57, 61, 62). In humans, high levels of CD23 are constitutively expressed mainly on naïve IgD+ B cells (44) and have been described to bind both free and monomeric IgE as well as differently composed and sized IgE-allergen complexes (63). Surface density of CD23 on B cells positively correlates with total serum IgE levels and determines activation of allergen-specific T cells upon loading with IgE-allergen complexes (44). Though affinity is one factor driving complex formation of allergen with IgE, clonality of the IgE repertoire is also an important factor in formation of complexes (64). The latter was especially important if IgE exhibited low to medium affinity for the allergen. While FcεRI-mediated internalization by dendritic cells seems to play a role in atopic dermatitis (59), CD23-mediated FAP by non-cognate B cells is an important mechanism in driving allergic rhinitis (44, 57, 65). The importance of CD23 in mediating allergen-presentation in the context of allergy is also supported by the fact that application of the anti-CD23 monoclonal antibody lumiliximab to allergen-stimulated PBMC cultures reduced their proliferation to allergen *in vitro* by 50% (66). In addition reduced IgE levels were observed upon anti-CD23 treatment in cell culture models suggesting a potential role of CD23 in regulating IgE production (67). Furthermore, inhibition of FAP by immunotherapy induced IgG is one important mechanism for the reduction of allergen-specific T cell and cytokine responses during AIT (68) as discussed below in more detail.

### Possible Mechanisms by Which Polyclonal Allergen-Specific IgG May Augment Activation of Effector Cells as Well as IgE^+^ B Cell Receptor-Bearing B Cells

**Figure 3:**
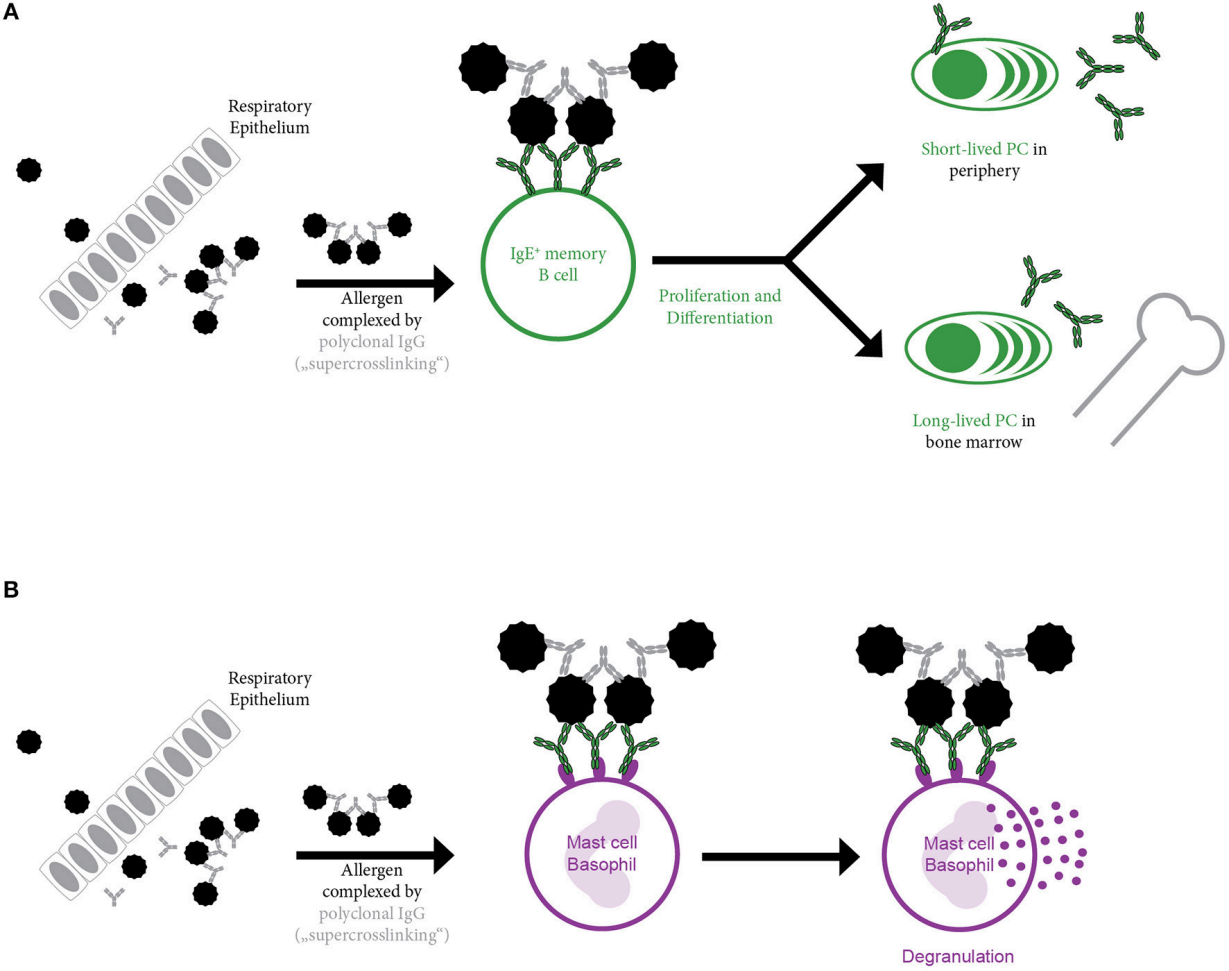
Super-crosslinking of allergen by allergen-specific polyclonal IgG. Upon entry e.g., through the epithelium, allergen is complexed by allergen-specific polyclonal IgG. The resulting multimeric allergen complex is able to **(A)** induce B cell activation by crosslinking membrane bound IgE present in form of the B cell receptor (BCR) on the surface of IgE^+^ memory B cells. This may result in activation of memory B cells in the absence of cognate T cell help and their differentiation into short-lived and long-lived plasma cells or may **(B)** induce basophil or mast cell activation by cross-linking of the FcεRI bound IgE molecules.

The absolute requirement of cognate T cell help for boosting an established allergen-specific IgE response has been challenged by several recent findings. In mice, using oligomeric B cell epitope-derived peptides for immunization in the absence of any adjuvants, a boost in allergen-specific IgE responses in the absence of any detectable allergen-specific T cell response was observed (69). Furthermore, blockage of co-stimulation capable of inhibiting the primary allergic sensitization of mice had no effect on re-challenge in an already established allergen-specific immune response (70). But also various observations in human subjects suggest that secondary IgE responses may not necessarily require T cell help. In this respect, nasal challenge only with the B cell epitope-containing complete allergen but not with fragments of the major birch pollen allergen Bet v 1 containing only T cell epitopes in the absence of B cell epitopes induced a relevant change of allergen-specific IgE levels (71). Interestingly, using a multicolour flow cytometry approach in combination with sequencing, the presence of two different IgE^+^ memory B cell subsets was suggested (41): One population with high replication history and high somatic hypermutation frequency similar to memory cells derived from primary immune responses. The second population showed a limited replication history as well as low somatic hypermutation levels reminiscent of a germinal center independent response. Finally yet importantly, in patients undergoing long-term systemic high-dose therapy with cyclosporine—that is known to suppress T cell responses—IgE levels for inhaled allergens as well as kinetics upon seasonal allergen exposure were not impaired (72). In this line, a similar result was obtained in HIV infected patients (73): Despite severely compromised CD4^+^ cell counts, patients suffering from AIDS showed a rise in IgE levels upon seasonal allergen exposure. Thus, it is conceivable that memory IgE^+^ B cells can also be activated solely upon antigen binding. Surpassing of the signaling threshold necessary for activation may e.g., be achieved by deposition and presentation of aggregated intact allergen or allergen-Ig complexes on the surface of follicular dendritic cells. However, it is also possible that soluble allergen itself is bound by polyclonal allergen-specific IgG and thus able to crosslink IgE^+^ B cell receptors (BCRs) upon binding. This potential mechanism of further oligomerizing IgE-allergen-complexes by allergen-specific IgG may be called “super-crosslinking” and obviously will depend on and vary with the concentrations, affinities and specificities of allergen-specific IgG (Figure 3A). It has previously been described in the context of mast cells where only super-crosslinking of IgE-monomer complexes with allergen-specific IgG induced relevant mediator release (Figure 3B) (74). Several other mechanisms for the enhancement of allergen-induced mast cell and/or basophil activation are possible. Effects which are independent of allergen-specific IgG are the occurrence of repetitive IgE epitopes on allergens and the occurrence of allergens as oligomers presenting the same epitope in a repetitive manner (75–77). For the major allergen of birch, Bet v 1, the occurrence of “catalytic” IgG antibodies has been reported which could enhance IgE binding most likely by exposing additional IgE epitopes by inducing a conformational change in the allergen (78–80). Bet v 1-specific “catalytic” IgG antibodies were even reported to enhance allergen-induced skin inflammation (81) and were considered as predictors for poor outcome of AIT (82). Alternatively, crosslinking of the IgE^+^ BCR may also be achieved by monomeric allergen itself as crystallization studies suggested (83). Using Phl p 7 as a model allergen, it was shown that IgE binding occurred in the classical way via the complementary determining regions and, in addition, that IgE was able to bind simultaneously to a different epitope of the allergen via residues in the V-domain framework regions, mimicking the mechanism of “superantigens.” These dual binding modes could lead to the cross-linking of IgE^+^ BCR in the absence of allergen-specific IgG antibodies. There are therefore several potential mechanisms by which T cell-independent B cell activation may occur but these need to be studied in greater detail in experimental models and evaluated for their relevance for allergy in humans.

## Effects of AIT-Induced Blocking Antibodies on Secondary Immune Responses to Allergen

The presence of an allergen-specific “inhibiting or immune type of substance” in post immunotherapy sera was first reported by Cooke and colleagues in 1935 (Figure 1) (84). They passively transferred serum obtained from patients before or after ragweed immunotherapy which had been mixed with the culprit allergen into the skin of non-sensitized subjects. They observed a reduced skin reaction if they applied serum from post AIT together with the allergen compared to pre-AIT allergen mixes. Subsequently, these thermostable antibodies were shown to compete for binding to the allergen with IgE, to reside in the IgG fraction and were termed “blocking” antibodies (26, 85). The pivotal role of blocking antibodies in successful AIT is emphasized by findings from a recent clinical trial where a single application of two blocking human monoclonal IgG antibodies specific for the major cat allergen Fel d 1 reduced symptoms upon nasal allergen challenge (86). The magnitude of reduction in symptoms upon a single passive immunization was similar to the one observed after years of conventional AIT.

## Reduced Effector Cell Activation in AIT-Treated Patients

**Figure 4:**
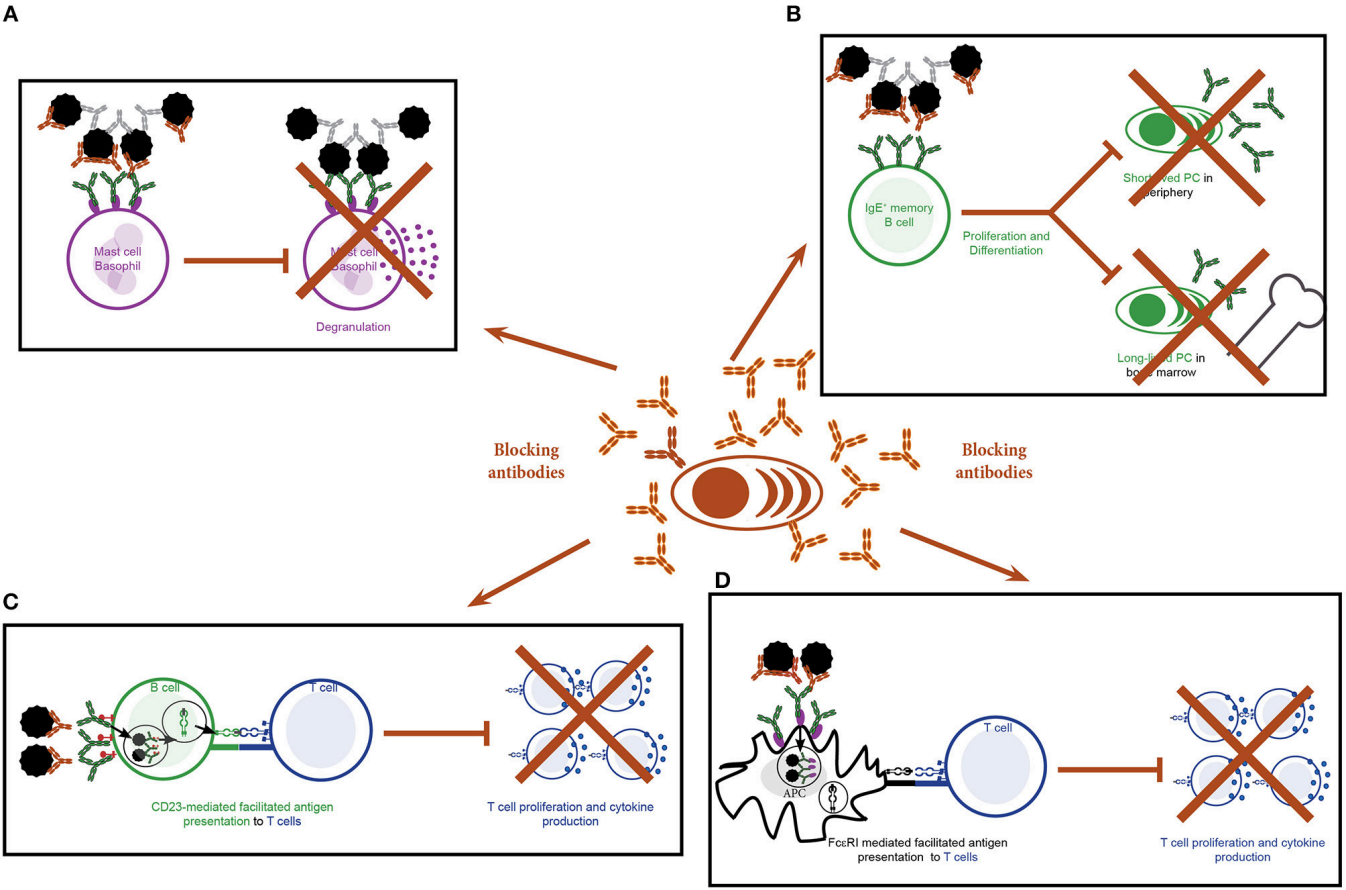
Effects of blocking antibodies induced by specific immunotherapy. **(A–D)** Production of allergen-specific IgGs by plasma cells targeting the IgE-binding sites of the allergen as blocking antibodies may have the following effects: **(A)** Blocking of allergen-induced activation of mast cells and basophils. **(B**) Blocking of binding of the allergen to the B cell receptor on IgE^+^ memory B cells and consequently inhibition of activation and differentiation of the cells. **(C)** Blocking of CD23-mediated, IgE-facilitated antigen presentation by B cells to T cells and hence T cell activation and differentiation. **(D)** Blocking of FcεRI-mediated, IgE-facilitated antigen presentation by B cells to T cells and hence T cell activation and differentiation.

Allergen binding to IgE bound to FcεRI on the surface of mast cells and basophils induces cross-linking of the receptors and hence activation of these cells (5). The interaction between allergen and IgE can be blocked by AIT-induced allergen-specific IgGs and consequently activation of effector cells is inhibited (Figure 4A). Numerous studies show a reduction in the activation of blood-derived basophils upon stimulation with allergen co-incubated with post but not pre-AIT sera (17, 87–90). Allergen-specific IgGs are not only detectable in serum but also in nasal secretions of SIT treated patients (20, 21). Here they will also reduce mast cell activation: Upon nasal challenge with allergen, AIT treated patients show reduced histamine, tosyl L-arginine methyl ester-esterase and kinins levels in nasal fluids (91). This was associated with a beneficial effect on nasal symptoms. In this respect, an association between levels of therapy-induced Bet v 1-specific IgG antibodies and reduced nasal sensitivity to the allergen was observed using genetically modified Bet v 1 derivatives for treatment (21). In addition to blocking binding of allergen to IgE, IgG antibodies are also thought to modulate mast cell responses by stimulation of inhibitory circuits upon binding to FcγRIIA and FcγRIIB receptors on mast cells (92).

### Blunting of Rises in Allergen-Specific IgE Levels Induced by Seasonal Allergen Exposure in AIT Treated Patients

Despite an initial transient rise in IgE levels upon start of subcutaneous immunotherapy (SCIT) with extracts, the allergen-specific IgE increase observed after seasonal natural pollen exposure is blunted during SCIT (31, 93–95). To surpass the disadvantages of conventional SCIT, efforts during the last decades focused on developing alternative approaches to achieve shorter treatment regimens as well as to reduce the risk of serious adverse side effects. To reduce the number of AIT vaccinations, new adjuvants strategies such as co-engagement of toll like receptors by conjugation of pollen allergens to monophoshoryl lipid A (88) or phosphorothioate oligodeoxyribonucleotide immunostimulatory DNA sequences (96) have been investigated in clinical trials. These studies also confirmed reduced seasonal augmentation of allergen-specific IgE levels observed during conventional AIT. In this line, also hypoallergenic derivatives of birch pollen have been shown to induce protective IgG antibodies that inhibited seasonal allergen-specific IgE rises as well allergen-induced effector cell responses (97). To engineer a vaccine that raises protective IgG antibodies but at the same time reduces both IgE- and T cell-mediated side effects, non-allergenic peptides from the IgE binding sites of major grass pollen allergens have been fused to the hepatitis B pre S protein (98, 99). After only three pre-seasonal vaccinations, a blunting of the seasonal allergen-specific IgE rise compared to the placebo group was achieved (100). Again, this coincided with the occurrence of allergen-specific IgG antibodies of the IgG_1_ and IgG_4_ subclass. Interestingly, this vaccine did also not induce an initial rise of IgE levels as observed during allergen extract-based SCIT and SLIT.

As an alternative to subcutaneous application, allergen extract can also be applied in a sublingual form (SLIT). However, direct comparison of SLIT and SCIT vs. placebo for a period of 3 years did not reveal any effect of SLIT on seasonal changes in allergen-specific IgE levels in contrast to SCIT (101).

Taken together SCIT leads to diminished rises in allergen-induced IgE levels induced by seasonal allergen exposure. This is most likely due to decreased IgE production. The latter can be explained by SCIT-induced blocking IgGs which inhibit binding of the allergen to the IgE^+^ BCR and thus reducing the activation of memory IgE^+^ B cells (Figure 4B) (3, 4).

### Reduced T Cell-Mediated Late Phase Responses in AIT- and Omalizumab-Treated Patients

Successful AIT is also associated with a reduction in the T cell-mediated late phase responses in target organs of allergy such as the skin, lung and nose (11, 14, 15, 102–104). In nasal mucosa of AIT-treated allergic patients the seasonal influx of CD3+ T cells is reduced (105) and nasal fluids bear reduced Th2 cytokine levels upon allergen challenge (106). Alongside with the decrease in IL-4 expressing cells there is an increase in the ratio of IFN-γ:IL-5 mRNA expressing cells (102, 103, 105). These effects coincide with the rise in blocking IgG antibodies and therefore blocking of IgE-mediated FAP is likely to account at least for part of the observed effect on T cells.

Blocking of IgE-facilitated antigen presentation by complexing allergen with serum IgG from patients undergoing birch pollen SIT was first demonstrated by van Neerven and co-workers (Figures 1, 4C,D) (27). Since then numerous studies confirmed that AIT induced IgGs block binding of IgE-allergen-complexes to CD23, a process called IgE-facilitated antigen binding (FAB) (Table 1) (12, 22, 27, 65, 106–119, 125–127). Among the IgG subclasses, FAB blocking ability is mostly attributed to allergen-specific IgG_4_ antibodies. IgG_1_ may have the same effects but this has not been studied in depth (112). Though the inhibition of FAB diminishes slowly after end of AIT treatment, inhibition of CD23-mediated allergen binding to B cells can be observed up to 2 years after discontinuation of immunotherapy (12, 22, 106, 110, 117). Interestingly, it has been observed that this persisting effect on FAB was not necessarily associated with the levels of allergen-specific IgG but may also depend on the avidity (22). Thus, it seems that the inhibitory bioactivity rather than the absolute levels of IgGs play a role in maintaining inhibition of FAB. Despite the FAB assay being a relatively fast and easy way of determining blocking activity of antibodies it bears one main limitation: It measures blocking of binding of an indicator serum derived from a patient with high allergen-specific IgE levels to a B cell line expressing high levels of CD23. Therefore, it may be difficult to directly translate these findings into potential effects of IgG on FAP *in vivo* for a given patient. However, in order to investigate the effect of blocking IgG on FAP in the treated patients a modification of the assay has been developed which allows to assess the direct blocking effect of immunotherapy-induced IgG antibodies on T cell proliferation *in vitro* for each of the treated patients (87). In the study describing the modified FAP assay, allergens were pre-incubated with sera derived from patients pre or post treatment with SIT or placebo before adding them directly to PBMCs cultures derived from the autologous patient. Using this model, a significant reduction in T cell proliferation in patients undergoing AIT- compared to placebo-treated controls was observed and confirmed the importance of IgG blocking antibodies in inhibiting FAP. However, it must be born in mind that so far the results of such *in vitro* FAP assays has not been associated with clinical parameters of late phase allergic inflammation and it is has therefore not been shown that the assay can be used as a surrogate for clinical outcome of AIT. The same is true for *in vitro* assays measuring the blocking of allergen-induced basophil activation by AIT-induced IgG.

**Table 1:** Effects of allergen-specific immunotherapy and anti-IgE (i.e., omalizumab) treatment on T cells in clinical trials in humans.

**Allergy to**	**Vaccination/intervention**	**Study design**	**Number of patients**	**Observation period**	**Outcome**	**Ref**.
**IMMUNOTHERAPY**						
Bee venom	SCIT with bee venom Alutard SQ	s.c. rush immunotherapy study	During SCIT (*n* = 10) After WD (*n* = 7)	3 y SCIT2 y after WD	Bee venom SCIT: Inhibition of FAB ↑ After WD: no diff to pre-treatment	(107)
Birch pollen	SCIT with Birch or Grass Alutard SQ	s.c immunotherapy, controlled study	Birch SCIT (*n* = 14) Grass SCIT (*n* = 2) Birch allergic (*n* = 7) Non-Allergic (*n* = 2)	3 to 30 m	Birch SCIT: Inhibition of FAP ↑, T cell line: ↓IL-4, IL-5, IL-10, IFN-γ, Inhibition of FAP by blocking antibodies	(27)
Birch pollen	SCIT with Birch Alutard SQ	Randomized, double-blind placebo-controlled study	Birch SCIT (*n* = 21) Placebo (*n* = 21)	18–20 m	Birch SCIT: Inhibition of FAP ↑, Association between inhibition of FAP and reduction of Bet v 1- specific IgE/IgG4 ratio	(65)
Birch pollen	SCIT with Birch Alutard SQ	Randomized, double-blind placebo-controlled study	Birch SCIT (*n* = 21) Placebo (*n* = 21)	18–20 m	Birch SCIT: Inhibition of FAB and FAP ↑ (1y>2y SCIT) Correlation of change in FAB and T cell proliferation	(108)
Birch pollen	SCIT with Bet v 1 trimer or fragments	Randomized, double-blind placebo-controlled study	Bet v 1 trimer (*n* = 14) Bet v 1 fragments (*n* = 11) Placebo (*n* = 21)	5 m	Birch SCIT: Inhibition of FAB ↑ trimer>fragments (n.s.) Correlation of Bet v 1 spec IgG with degree of inhibitory activity in FAB	(109)
Birch Pollen	Birch ALK Depot SQ	s.c. immunotherapy study	Birch SCIT (*n* = 16)	3 y SCIT1.5 y after WD	Birch SCIT: Inhibition of FAB↑ maximum at 3y SCIT, still inhibition 1.5 y after WD Th2 response PBMC cultures: ↓ 4.5 y	(110)
Grass pollen	SCIT with Grass Alutard SQ	Randomized, double-blind placebo-controlled study	Grass SCIT (*n* = 10) Placebo (*n* = 8)	1 y	Grass SCIT: Inhibition of FAB and FAP ↑ Inhibition of FAB mediated by IgG Correlation of degree of allergen-IgE binding to B cells and T cell prolif	(111)
Grass pollen	SCIT with Grass Alutard SQ	Double-blind placebo-controlled study	Grass SCIT (*n* = 20) Placebo (*n* = 17)	2 y	Grass SCIT: Inhibition of FAB ↑ after 2y SCIT IgG4 fraction mediates inhibition of FAB	(112)
Grass pollen	SCIT with Grass Alutard SQ	Randomized, double-blind placebo-controlled study	Grass SCIT (*n* = 12) Placebo (*n* = 6)	1 y	Grass SCIT: Inhibition of FAB ↑ 6 weeks until 1 y SCIT Smaller early and late phase SPT responses	(113)
Grass pollen	SLIT with Soluprick SQ	Randomized, double-blind placebo-controlled study	Grass SLIT (*n* = 14) Placebo (*n* = 9) Non-atopic control (*n* = 8)	12–18 m	Grass SLIT: Inhibition of FAB ↑ at 3 months and 15 months	(114)
Grass pollen	SCIT with Grass Alutard SQ	Randomized, double-blind placebo-controlled discontinuation study	Grass SCIT 4 y (*n* = 7) Grass SCIT/Placebo 2 y/2 y (*n* = 6)	4 y SCIT2 y SCIT/2y Placebo	Grass SCIT: Inhibition of FAB ↑ also after 2y discontinuation Inhibition of FAB dependent on IgG4. IgG4 ↓ after end of SCIT but still inhibition of FAB ↑ Inverse correlation of magnitude of inhibition of FAB and SMS	(22)
Grass pollen	SCIT with Grass Alutard SQ	Dose-response randomized double-blind placebo- controlled study	100 000 SQU (*n* = 112) 10 000 SQU (*n* = 53) Placebo (*n* = 55)	8 m	Grass SCIT: Inhibition of FAB ↑ 100 000 SQU > 10 000 SQU Modest inverse correlation of change in FAB and combined SMS	(115)
Grass pollen	SLIT with ALK Grazax	Randomized, double-blind placebo-controlled study	Grass SLIT (*n* = 316) Placebo (*n* = 318)	3 y SLIT2 y WD	Grass SLIT: Inhibition of FAB ↑ max after 3y then decline but still present after 2 y WD	(12)
Grass pollen	SCIT with Grass Alutard SQ SLIT with ALK Grazax	Randomized, controlled study	Grass SCIT (*n* = 15) Grass SLIT (*n* = 15) Control group (*n* = 10)	15 m	Grass AIT: FAB inhibition at 3 months: ↑ SCIT > SLIT FAB inhibition at 12 months: ↑ SCIT = SLIT	(116)
Grass pollen	SCIT with Grass Alutard SQ SLIT with ALK Grazax	Controlled s.c./SLIT study	SCIT (*n* = 14) SLIT (*n* = 12) SLIT/WD 3 y/1–2 y (*n* = 6) SAR (*n* = 24) Non-atopic (*n* = 12)	1–3 y AIT1–2 y WD	Grass AIT: Inhibition of FAB ↑: SCIT > SLIT/WD > SLIT	(117)
Grass pollen	SCIT with recombinant B cell epitope-based vaccine BM32	Randomized, double-blind placebo-controlled study	40 μg BM32 (*n* = 17) 20 μg BM32 (*n* = 18) 10 μg BM32 (*n* = 17) Placebo (*n* = 18)	19 w	Grass SCIT: ↓ T cell prolif to mix of recombinant Phl p 1, 2, 5, 6 in presence of post-SCIT serum	(87)
Grass pollen	SCIT with Lolium perenne peptides (LPP)	Dose escalation study	SCIT (*n* = 61)	8 w	Grass SCIT: Inhibition of FAB ↑ after 5 and 8 weeks	(118)
Grass pollen	SCIT with Grass Alutard SQ SLIT with ALK Grazax	Randomized, double-blind placebo-controlled study	SLIT/Placebo SCIT (*n* = 36) Placebo SLIT/SCIT (*n* = 36) Placebo SLIT/Placebo SCIT (*n* = 34)	2 y AIT1 y WD	Grass AIT: Inhibition of FAB ↑ for 2y IT and 1y WD (SLIT = SCIT) ↓Frequency of Ag-specific Th2 only for 2y IT (not for 1y after WD)	(106)
Grass pollen	SCIT with LPP with/without DnaK	Randomized, double-blind placebo-controlled study	LPP (*n* = 9) LPP/DnaK (*n* = 9) Placebo (*n* = 9)	25 w	Grass AIT: Inhibition of FAB ↑at w25 in LPP group only	(119)
**OMALIZUMAB**						
Cat Dander	Omalizumab s.c.	Randomized, double-blind placebo-controlled study	Oma (*n* = 12) Placebo (*n* = 4)	15 w	Omalizumab: T-DC co-cultures post-treatment: 20-40% ↓ allergen-spec T cell prolif; ↓IL-5, IL-13, IL-10 in supernatants	(120)
N.A.	Omalizumab s.c.	Randomized, double-blind placebo-controlled study	Oma (*n* = 18) Placebo (*n* = 17)	60 w	Omalizumab: Serum: ↓ levels of IL-13 (*p* < 0.01), IL-5 (trend but ns) 16 weeks after therapy	(121)
N.A.	Omalizumab, s.c.	Randomized, double-blind placebo-controlled study	Oma (*n* = 14) Placebo (*n* = 14)	4 m	Omalizumab: In submucosa of bronchial biopsies: ↓CD3, CD4, CD8 cells, IL-4 and IgE	(122)
N.A.	Omalizumab s.c.	Randomized, double-blind placebo-controlled study	Oma (*n* = 12) Placebo (*n* = 12)	12 w	Omalizumab: Skin biopsy after intradermal allergen challenge: ↓Late phase reaction >> early phase reaction	(123)
N.A.	Omalizumab, s.c.	Randomized, double-blind placebo-controlled study	Oma (*n* = 9) Placebo (*n* = 10)	12 w Oma12 w WD	Omalizumab: In blood: ↓ IL-2+CD3+, IL-13+CD3^*^, CMSF+CD3+ lymphocytes after 12w treatment	(9)
Ragweed Pollen	Omalizumab s.c. Aqueous short ragweed extract ALK-Abello s.c.	Randomized, double-blind placebo-controlled study	Oma/SCIT (*n* = 7) Oma/Placebo (*n* = 11) Placebo/SCIT (*n* = 9) Placebo/Placebo (*n* = 9)	64 w	Omalizumab/SCIT: During Treatment Inhibition of FAB ↑: Oma/SCIT or Oma/Placebo >> SCIT End study: Inhibition of FAB↑ only in Oma/SCIT group	(124)

The hypothesis that allergen-specific IgE levels influence T cell responses is supported by findings from therapeutic strategies directly targeting and thus reducing serum IgE levels such as omalizumab. Omalizumab is a monoclonal anti-IgE antibody binding to the constant region 3 of IgE and hence inhibits binding of IgE to its receptors FcεRI and CD23 (128, 129). Treament with omalizumab reduces levels of free serum IgE by 95% and leads to reduced exacerbations and corticosteroid use in asthmatic patients (130–132). Interestingly, it also attenuates late phase responses upon allergen challenge in lung as well as in skin of atopic subjects (7, 123) (Table 1). In this context it has been reported that there is a reduction in IL-13+ CD3+ lymphocytes (9) as well as in IL-13 levels (121) in the blood of omalizumab-treated patients. But the effect of omalizumab is also evident locally in target organs of allergy such as the lung: Diminished levels of CD3, CD4, CD8 lymphocytes as well as reduced staining for IgE and IL-4 were observed in the submucosa of bronchial biopsies of omalizumab-treated patients (122). All these aforementioned results indicate that the decrease in free IgE levels upon omalizumab treatment also influences IgE-facilitated allergen presentation via IgE receptors and subsequent T cell stimulation (Table 1). In this respect, allergen-induced proliferation in DC/T co-cultures was reduced by 20–40% in patients undergoing treatment with omalizumab (120).

The observation that there is a cumulative effect of both AIT and omalizumab treatment on FAB especially after discontinuation of the treatment (124) supports the hypothesis that both allergen-specific IgE and IgG antibodies are important in regulating allergen-specific T cell responses.

In addition to producing antibodies, a subset of IL-10 producing regulatory B cells, the so called B_R_1 cells, may also exert immunomodulatory effects on T cell proliferation in allergic responses (133). This population of B cells was isolated from the blood of patients undergoing bee venom immunotherapy and is characterized by high surface expression of CD25 and CD71 as well as low expression of CD73 (134). In this study B_R_1 cells were shown to both suppress antigen-specific T cell proliferation in an IL-10 dependent manner and to selectively upregulate IgG_4_ production upon differentiation into plasma cells. However, the importance of B_R_1 and other regulatory B cell subsets in mediating immunomodulatory effects in the context of immunotherapies targeted inhalant allergens warrants further investigation. Furthermore, the antigen-specificity of the IgG_4_ responses needs to be investigated.

## Conclusion

There is no doubt, that allergen-specific T cell help is critically required for the establishment of an allergen-specific IgE response and the process called allergic sensitization (i.e., primary allergic immune response) which leads to the establishment of an allergic immune response. However, there is compelling evidence that once allergic sensitization has occurred in an allergic individual, the secondary allergen-specific IgE and T cell response is regulated by allergen-specific IgE and IgG antibodies. It has been demonstrated that allergen-specific IgG antibodies which are for example induced during AIT and are directed against the IgE binding sites of allergens can prevent allergen-induced immediate and delayed allergic inflammation as well as specific IgE production. This can be achieved by inhibition of allergen-induced cross-linking of IgE bound to high affinity receptors for IgE on mast cells and basophils, by inhibition of IgE-facilitated allergen presentation to T cells and by preventing boosts of allergen-induced IgE production by IgE memory B cells by blocking IgG antibodies. Much less is known about the potential role of allergen-specific IgG in enhancing allergen-specific immune responses but it is possible that under certain conditions allergen-specific IgG may super-crosslink allergen-IgE immune complexes on B cells, mast cells, basophils and eventually APCs and thus contribute to boosting of secondary allergen-specific IgE production in the absence of T cell help, to the enhancement or blocking of allergen-induced basophil and mast cell activation and may also have as yet unknown effects on IgE-facilitated allergen presentation and T cell activation. The role of the latter mechanisms and their biological effects need further investigations.

## Author Contributions

JE-D, SV-M, BL, and RV wrote the manuscript. JE-D, RV and SV-M designed the Figures and Table. AK, YZ, MK, and VN-L critically read and revised the manuscript.

### Conflict of Interest Statement

RV has received research grants from Biomay AG and Viravaxx, Vienna, Austria and serves as a consultant for these companies. The remaining authors declare that the research was conducted in the absence of any commercial or financial relationships that could be construed as a potential conflict of interest.

## Abbreviations

Ab: antibody
APC: antigen presenting cell
BCR: B cell receptor
B_R_1: B regulatory cell 1
CD: cluster of differentiation
FAB: facilitated antigen binding
FAP: facilitated antigen presentation
HIV: human immunodeficiency virus
AIDS: acquired immunodeficiency syndrome
Ig: immunoglobulin
IL: interleukin
SCIT: subcutaneous immunotherapy
AIT: allergen-specific immunotherapy
SLIT, sublingual immunotherapy FAB: facilitated antigen binding
FAP: facilitated antigen presentation
LPP: *Lolium perenne* peptide
DnaK: prokaryotic heat shock protein
m: month
N.A.: not applicable
Oma: Omalizumab
SAR: seasonal allergic rhinitis
s.c.: subcutaneous
SCIT: subcutaneous immunotherapy
SLIT: sublingual immunotherapy
SIT: specific immunotherapy
SMS: symptom medication score
WD: withdrawal
y: year.
